# The effectiveness of a government-sponsored health protection scheme in reducing financial risks for the below-poverty-line population in Bangladesh

**DOI:** 10.1093/heapol/czad115

**Published:** 2023-12-20

**Authors:** Md Zahid Hasan, Sayem Ahmed, Gazi Golam Mehdi, Mohammad Wahid Ahmed, Shams El Arifeen, Mahbub Elahi Chowdhury

**Affiliations:** Health Systems and Population Studies Division, icddr,b, Dhaka 1212, Bangladesh; Academic Unit of Health Economics, Leeds Institute of Health Sciences, University of Leeds, Worsley Building, Clarendon Way, Leeds LS2 9NL, UK; Nuffield Centre for International Health and Development, Leeds Institute of Health Sciences, University of Leeds, Worsley Building, Clarendon Way, Leeds LS2 9NL, UK; Health Economics and Health Technology Assessment, School of Health and Wellbeing, University of Glasgow, 90 Byres Road, Glasgow G12 8TB, UK; Centre for Health Economics and Medicines Evaluation, Bangor University, Ardudwy, Normal Site, Holyhead Road, Gwynedd, Wales LL57 2PZ, UK; Health Systems and Population Studies Division, icddr,b, Dhaka 1212, Bangladesh; Health Systems and Population Studies Division, icddr,b, Dhaka 1212, Bangladesh; Maternal and Child Health Division, icddr,b, Dhaka 1212, Bangladesh; Health Systems and Population Studies Division, icddr,b, Dhaka 1212, Bangladesh

**Keywords:** Below-poverty-line population, health protection scheme, catastrophic healthcare expenditure, out-of-pocket payment, impoverishment, Bangladesh

## Abstract

The Government of Bangladesh is piloting a non-contributory health protection scheme called Shasthyo Surokhsha Karmasuchi (SSK) to increase access to quality essential healthcare services for the below-poverty-line (BPL) population. This paper assesses the effect of the SSK scheme on out-of-pocket expenditure (OOPE) for healthcare, catastrophic health expenditure (CHE) and economic impoverishment of the enrolled population. A comparative cross-sectional study was conducted in Tangail District, where the SSK was implemented. From August 2019 to March 2020, a total of 2315 BPL households (HHs) (1170 intervention and 1145 comparison) that had at least one individual with inpatient care experience in the last 12 months were surveyed. A household is said to have incurred CHE if their OOPE for healthcare exceeds the total (or non-food) HH’s expenditure threshold. Multiple regression analysis was performed using OOPE, incidence of CHE and impoverishment as dependent variables and SSK membership status, actual BPL status and benefits use status as the main explanatory variables. Overall, the OOPE was significantly lower (*P* < 0.01) in the intervention areas (Bangladeshi Taka (BDT) 23 366) compared with the comparison areas (BDT 24 757). Regression analysis revealed that the OOPE, CHE incidence at threshold of 10% of total expenditure and 40% of non-food expenditure and impoverishment were 33% (*P* < 0.01), 46% (*P* < 0.01), 42% (*P* < 0.01) and 30% (*P* < 0.01) lower, respectively, in the intervention areas than in the comparison areas. Additionally, HHs that utilized SSK benefits experienced even lower OOPE by 92% (*P* < 0.01), CHE incidence at 10% and 40% threshold levels by 72% (*P* < 0.01) and 59% (*P* < 0.01), respectively, and impoverishment by 27% at 10% level of significance. These findings demonstrated the significant positive effect of the SSK in reducing financial burdens associated with healthcare utilization among the enrolled HHs. This illustrates the importance of the nationwide scaling up of the scheme in Bangladesh to reduce the undue financial risk of healthcare utilization for those in poverty.

Key messages

- The government-initiated social health protection scheme, Shasthyo Surokhsha Karmasuchi (SSK) has been introduced primarily for the below poverty line (BPL) population and only for seeking inpatient care from designated government hospital.
- Overall, the out-of-pocket expenditure (OOPE), the incidence of catastrophic health expenditure (CHE) and impoverishment were significantly lower (33%, 46% and 30%, respectively) in the areas covered by the SSK vs the comparison areas.
- The SSK members who utilized inpatient care services had 92% lower OOPE and 72% lower incidence of CHE than the comparison areas.
- A clear strategy should be taken to identify the BPL households and a rigorous enrolment process should be followed for the effective utilization of such scheme.

## Introduction

The Bangladesh National Health Accounts for 2015 revealed that the share of out-of-pocket expenditure (OOPE) of households (HHs) in the total healthcare expenditure increased from 63% in 2012 to 68.5% in 2020 (MOHFW, 2022). A recent study showed that overall, 24.6% of HHs in Bangladesh incurred catastrophic health expenditure (CHE) due to OOPE for healthcare in 2016. This study estimated that 4.5% of the total population fell into poverty due to such high OOPE for healthcare, resulting in the economic impoverishment of 8.61 million people annually (Ahmed *et al.*, 2022). To achieve universal health coverage, the World Health Organization urges member states to establish an affordable healthcare financing system; this includes a prepayment healthcare financing scheme that allows sharing of the risk of high healthcare OOPE among the population and avoidance of related CHE and impoverishment (WHO, 2005). Under Sustainable Development Goal 3.8, Universal Health Coverage promises access to quality essential healthcare services without the risk of financial hardship (United Nations, 2015). Health insurance schemes serve as prepayment risk-pooling mechanisms, enabling enrolled members to access need-based healthcare from designated providers without suffering unforeseen or unaffordable healthcare costs (Cichon *et al.*, 1999).

Several low- and middle-income countries (LMICs) have adopted health insurance models as an affordable financing mechanism for healthcare that reduces reliance on OOPE (Kuwawenaruwa *et al.*, 2016; Karan *et al.*, 2017; Philibert *et al.*, 2017; Gyasi *et al.*, 2018). There are broadly two approaches of financing such health insurance model: (1) contributory health insurance, where benefits are linked to pre-payment in the form of mandatory or voluntary insurance premiums and enrolees get access to certain health services and (2) non-contributory health insurance, where benefits are not linked to contributions for health payment and are typically funded from general government budget revenue. In 2012, the Government of Bangladesh adopted a healthcare financing strategy with a view to bringing all citizens under financial protection by 2032 (MOHFW, 2012). To achieve this goal, a health protection scheme, Shasthyo Surokhsha Karmasuchi (SSK), has been developed by the Health Economics Unit (HEU), Ministry of Health and Family Welfare, of the Government of Bangladesh. Initially, the scheme is being piloted as a non-contributory scheme among the below poverty line population (BPL) in a selected district. The authorities have plans to gradually expand its coverage to include the remaining population (HEU; MOHFW, 2014).

Health insurance or health protection schemes have been evaluated in several countries to assess their effect on health service utilization, OOPE, financial risk protection and health status. Several studies have indicated that insurance improves utilization and reduces personal healthcare expenditure (Smith and Moreno-Serra, 2012; Ahmed *et al.*, 2018b; 2020; 2021b; Khan *et al.*, 2020). However, other studies have found a heterogeneous or null effect of insurance on healthcare utilization and related OOPE (Wagstaff *et al.*, 2009; Fink *et al.*, 2013). A systematic review of studies conducted in the LMICs on the impact of publicly financed health insurance schemes demonstrated a positive effect of insurance in the form of increased healthcare utilization among the insured population, improved financial protection and improved health status with some exceptions (Erlangga *et al.*, 2019). Two evaluation studies on the state-sponsored health insurance schemes in India have reported a decline in OOPE among enrolled HHs (Fan *et al.*, 2012; Sood *et al.*, 2014). Moreover, an evaluation of a community-based health insurance scheme in Bangladesh showed that utilization of healthcare from a medically trained provider significantly increased among the insured group while experiencing a lower OOPE compared with the uninsured group (Ahmed *et al.*, 2018b; Khan *et al.*, 2020). Finally, a study on employer-sponsored health insurance in Bangladesh reported a significant positive effect of the scheme in increasing healthcare utilization when given by a medically trained provider and a non-significant effect on the reduction of OOPE among them (Ahmed *et al.*, 2020).

### Shasthyo Surokhsha Karmasuchi

The SSK scheme was being piloted in three Upazilas (sub-districts) of Tangail District, namely Kalihati, Madhupur and Ghatail, to improve the BPL population’s access to inpatient care (IPC) hospital services. The pilot SSK scheme was initially launched in Kalihati in March 2016 and subsequently expanded to Madhupur and Ghatail Upazilas over the following 18 months. The SSK initially enrolled BPL population under the scheme and has plans to include the remaining population in the future (HEU; MOHFW, 2014). As of now, the scheme operates as a non-contributory system for the BPL population and individuals outside this category cannot purchase insurance from SSK authority.

Under the scheme, an HH is classified as a BPL HH if it meets any two of the following criteria: (1) The HH head is a regular day labourer, (2) the HH has no land, except for a dwelling place and (3) the HH has no permanent income source (Barkat *et al.*, 2012). A detailed description of the scheme is published elsewhere (Ahmed *et al.*, 2018a). In the three pilot Upazilas, the insured HHs receive healthcare services via 50- to 100-bed public hospitals called Upazila health complexes (UzHCs), one per Upazila, with a referral linkage with the district hospital (DH) situated at the district headquarters in Tangail. It is noted that healthcare services at public hospitals in Bangladesh are not entirely free and not all the prescribed drugs and diagnostic services are available or provided free of charge at these hospitals. However, under the SSK scheme, the insured patients get healthcare services from the public hospitals with some additional benefits. For instance, the scheme has contracted private pharmacies and clinics for providing prescribed drugs and diagnostic services to the insured inpatients that are not available at the hospitals. Non-insured patients seek healthcare from these hospitals without getting such benefits of the SSK scheme. In the case of outpatients, the insured patients only get free consultation while medicine and diagnostic services for insured outpatients are similar to those provided to the non-insured patients.

### Scheme management

The key actors in this initiative included HEU-formed SSK Cell, contracted scheme operator, the UzHCs and local-level SSK implementation committees (HEU; MOHFW, 2014). The SSK Cell worked as the key management body to implement the SSK scheme. This management body contracted Green-Delta Insurance Company Limited, a private insurance provider in the country, to act as the scheme operator. The scheme operator was responsible for various operational activities except providing healthcare. These responsibilities include enlisting BPL HHs, issuing health cards (an identity card for accessing the services of the SSK scheme), assisting cardholders in receiving healthcare services at selected hospitals, organizing claim documents, assisting the UzHCs and DH in claim submission and getting reimbursement and monitoring the scheme activities (HEU; MOHFW, 2014).

### Benefits packages

The non-contributory scheme offered healthcare to the identified BPL population for 78 disease groups or health conditions (Healthy DEvelopments, 2018). In SSK health facilities, insured patients received all prescribed drugs and diagnostic tests through contracted providers if these items are not available at the hospitals. Additionally, transportation services to the DH were provided to the insured patients for referral cases. The premium for this scheme was paid by the government at a rate of BDT 1000 per HH per year, with maximum financial protection of BDT 50 000 per HH per year. Based on the premium, a pool of funds is allocated within the operational plan of ‘Health Economics Unit’ for operating the scheme.

### Service delivery and financing

The hospitals (UzHCs and DH) provided healthcare services to the SSK cardholders without charging any user fees for them. There is no gatekeeping system below the UzHCs to eliminate direct patient access to hospital services. When an insured patient arrives at the SSK registration booth and presents the health card, the patient is sent to doctors’ room for consultation. The doctor diagnoses the patient and determines whether the patient requires hospital admission. If admission is required, the patient is admitted to the hospital under the SSK scheme; otherwise, he/she gets regular outpatient services at the hospital, similar to non-insured patients.

The expenditures for treating the patients are reimbursed to the hospitals by the SSK Cell within 30 days of claim submission. Reimbursements to the hospitals are made based on verifiable patient records (claims) following a case- and diagnosis-based payment system that was developed following diagnosis-related groups. There are 78 disease groups under which patients are treated at SSK hospitals. The hospitals get reimbursed a fixed fee for each disease group. Following reimbursement, the hospitals pay for the additional medicines and diagnostic services provided to the insured patients and can spend the remaining fund for improving hospital services. The service delivery and claim records are managed via a computerized hospital management system at the hospitals.

Although a previous study was conducted to identify the implementation-related challenges of the SSK health protection scheme in one Upazila (Ahmed *et al.*, 2018a), to this day, no comprehensive assessment has been undertaken to understand the SSK’s effectiveness in the reduction of financial risks and to identify the scheme’s implementation-related challenges. This research aims to contribute to the empirical evidence by examining how SSK scheme has affected healthcare-related financial risk of the BPL HHs by estimating the changes in OOPE, CHE incidence and impoverishment in the SSK intervention areas as compared with the comparison areas.

## Methods

### Study design and settings

**Figure 1.**
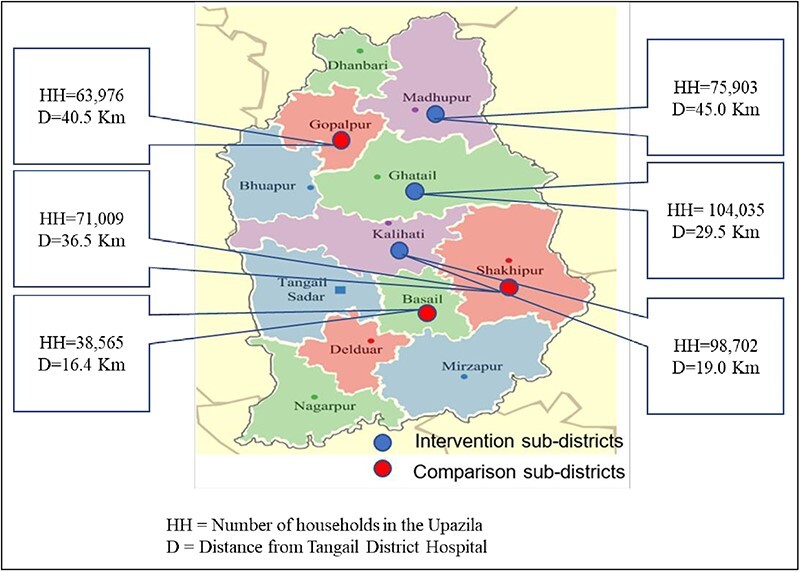
The Shasthyo Surokhsha Karmasuchi–implementing Upazilas in Tangail District and their distance from the district hospital

This cross-sectional comparative study used an intervention group and a comparison group to assess the SSK schemes’ effectiveness in reducing financial burden. The intervention group consisted of the BPL HHs in the three SSK intervention areas, i.e. Kalihati, Ghatail and Madhupur Upazilas of Tangail District. The comparison group comprised the identified BPL population in three equivalent comparison Upazilas, i.e. Basail, Gopalpur and Shokhipur of the same district. The comparison Upazilas were selected based on their comparative distance from each intervention Upazila to the Tangail DH, which served as a referral facility for all selected Upazilas (Figure 1).

### Sample selection process

In each of the intervention and comparison areas, the HHs were selected in two stages. In the first stage, for each Upazila, village stratification was performed considering the accessibility from the centre of each village to the UzHC of the respective Upazila. Information on the distance, travel time and costs from the centre of each village to the respective UzHC was collected through a village mapping survey. Applying principal component analysis on those parameters, the villages were classified into three strata of geographical accessibility, i.e. easy, medium and difficult. In the second stage, for each intervention and comparison Upazila, 10 villages were randomly selected from each accessibility stratum. Thus, for each intervention and comparison area, 30 villages were selected from each stratum of accessibility, and in total 90 villages were selected in each of the study areas (30 villages from each Upazila) (Appendix 1).

### Data collection

#### Household screening

A two-step screening process was used to identify eligible HHs for a detailed interview in both the intervention and comparison areas of a study. In the first step, a screening form was administered to identify the eligible HHs for a detailed interview after obtaining their informed consent. In intervention areas, an insured HH (identified as BPL by SSK programme) was eligible if it had at least one individual who had IPC in the last 12 months. Whereas, in comparison areas, an uninsured HH was eligible if it met BPL selection criteria and had at least one individual who had IPC in the last 12 months. The screening form consisted of two sections.

The first section assessed whether a visited HH met BPL selection criterion. HHs identified as BPL HHs in the intervention and comparison areas were those HHs that met the BPL selection criterion during the HH screening by the study team. In the intervention areas, insured HHs that did not met the BPL selection criterion were classified as non-BPL HHs, although these HHs were insured by the SSK scheme as they were listed as BPL during their enrolment. The second section of the screening form was used to identify whether the HH had at least one member who had sought IPC within the last 12 months. Additionally, information on the illness and hospitalization status of HH members was collected. In the intervention areas, both sections of the form were administered to a total 7158 SSK-identified BPL HHs from the selected villages. In the comparison areas, 29 793 HHs from the selected villages were screened using the same criteria of which 7886 (26.5%) HHs were identified as BPL. These BPL HHs then were administered the second part of the screening form to collect additional information.

#### Household survey

The data were collected through face-to-face interviews after obtaining informed consent with an adult respondent, preferably the head of the HH. In the absence of the head of the HH at the time of the data collector’s visit, a responsible adult member from that HH was selected for the interview. A total of 12 trained data collectors and two trained supervisors were recruited for this study who conducted the interviews in both the intervention and comparison areas from August 2019 to March 2020.

**Figure 2.**
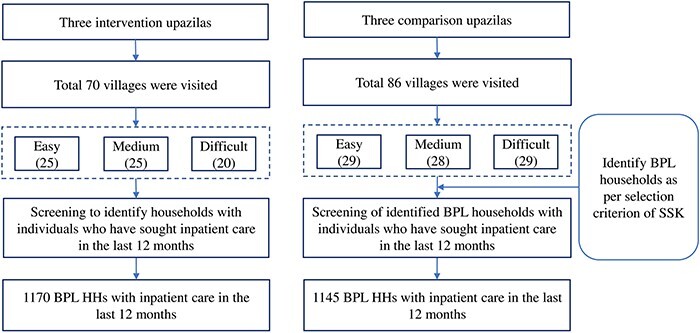
Sample selection criterion

Although 90 villages were selected in each intervention and comparison area, the data collectors visited only 70 villages (25 easy, 25 medium and 20 difficult) in the intervention areas and 86 villages (29 easy, 28 medium and 29 difficult) in the comparison areas **(**Figure 2). Of the 20 villages not covered in the intervention areas, 18 had no SSK-identified BPL HHs. Moreover, two villages in the intervention areas and four villages in the comparison areas could not be covered due to the start of the coronavirus 2019 pandemic. In the villages visited, the data collectors interviewed 1170 insured HHs in the intervention areas and 1145 HHs without SSK in the comparison areas. The number of HHs interviewed were more than minimum required sample of 795 HHs in each area. Along with the demographic and socioeconomic characteristics of the HH members, detailed information was collected concerning their experience with IPC, episode of illness by type, related healthcare-seeking and OOPE for healthcare. Information on the HH members’ healthcare seeking and OOPE for healthcare was collected for the prior 12 months in case of IPC utilization and for the prior 3 months in case of outpatient care utilization.

### Data analysis

The proportion of patients who utilized healthcare from different sources, the average OOPE for healthcare, the CHE incidence and impoverishment from OOPE for healthcare were estimated for both the intervention and comparison areas. In the intervention areas, the insured HHs were classified based on SSK benefits usage (i.e. utilized SSK benefits or did not utilize SSK benefits) as well as their current BPL status (i.e. BPL and non-BPL).

Regarding the OOPE, consultation, registration, medicine, diagnostics tests, hospital bed fees, surgical operations and packaged care (e.g. delivery) were considered as direct medical expenditures, while expenses for transportation, attendants and any other items (e.g. tips) were considered as direct non-medical expenditures (van Doorslaer *et al.*, 2006).

#### Estimation of catastrophic health expenditure

The CHE incidence was estimated and compared between the HHs in the intervention and comparison areas to assess the effect of SSK scheme on CHE among the SSK members. Furthermore, the CHE incidence was compared between the HHs that utilized the SSK services and those that did not utilize SSK services in the intervention areas. Two CHE thresholds were employed to assess whether a HH’s health spending was catastrophic: (1) 10% of total consumption expenditure as suggested by Wagstaff and van Doorslaer (2003), and (2) 40% of non-food consumption expenditure, a proxy measure of a HH’s capacity to pay as proposed by O’Donnell *et al.*, (2008) and Xu *et al.*, (2003) (Wagstaff and van Doorslaer, 2003; Xu *et al.*, 2003; O’Donnell *et al.*, 2008). The HHs with an OOPE exceeding either of the two thresholds were considered to have experienced CHE.

#### Estimation of impoverishment due to out-of-pocket expenditure for healthcare

The effect of OOPE for healthcare on the impoverishment of the HHs was estimated separately for the intervention and the comparison areas, and then the difference in impoverishment between the two areas was compared. The impoverishment was measured by applying a poverty line estimated for the study population following the cost of basic needs approach used by the Bangadesh Bureau of Statistics (2017). In this approach, the poverty line represented the level of per capita expenditure at which the members of an HH could expect to meet their basic needs (comprising food and non-food items). The market price for 11 food items (i.e. rice, wheat, pulses, milk, oil, meat, fish, potato, other vegetables, sugar and fruits), comprising 2122 kcal per day per person, was calculated to construct the food poverty line for the HHs (Ahmed *et al.*, 2017). Then, the non-food allowance for the poverty line was estimated as the median amount spent on non-food items by a group of HHs that had per capita food expenditure close to the food poverty line. The poverty line was the sum of the food poverty line and non-food allowance of the HHs.

Considering this poverty line, the proportion of HHs in the study areas that had been pushed into poverty due to OOPE for healthcare was measured. First, the poverty rate was estimated using the total consumption expenditure (including OOPE for healthcare). A HH was considered poor if its total consumption expenditure was lower than the poverty line. Second, the poverty rate was estimated using the total expenditures of HHs excluding any OOPE for healthcare. A difference between the two poverty rates (i.e. with and without OOPE) was considered as impoverishment due to OOPE for healthcare. This estimation was conducted separately for the intervention and comparison areas. The differences in poverty rates between the areas were then used to determine the effect of the SSK services on the HHs’ impoverishment due to OOPE for healthcare (Akazili *et al.*, 2017).

### Statistical analysis

We performed the chi-squared test for testing the relationship between socioeconomic characteristics and study group (e.g. intervention, comparison groups). We have conducted t-test to compare the mean differences between groups (e.g. mean monthly food expenditure between intervention and comparison groups). We used z-test of proportion to compare the mean CHE and impoverishment across groups. The OOPE showed a right skewed distribution (Supplementary Figure 1), and the Shapiro-Francia normality test was statistically significant in rejecting the hypothesis of normal distribution. Thus, we performed a non-parametric Wilcoxon rank-sum test to compare the difference of OOPE between different groups. A multiple linear regression model was applied to predict the relationship between OOPE and SSK membership status. In this model, the natural log transformation of OOPE was used as the dependent variable to reduce the effect of the skewed nature of the OOPE.

The model was specified as follows:


(1) $$ln{\ }({Y_{1i}}) = {\beta _0} + {\beta _1}{X_{1i}} + {\beta _2}{X_{2i}} + {\beta _3}{X_{3i}} + \ldots + {\varepsilon _i}{\ }$$

where β_0_ is a constant, *X_1_* indicates the study area with values 0 or 1 (0 = Comparison, 1 = Intervention), *β_1_* is the coefficient that shows the magnitude and direction of relationship, *X_2_, X_3_*, … denote control variables, *β_2_, β_3_* represent the estimated coefficients and *ε_i_* is the random error term of the model.

Furthermore, multiple binary logistic regression models were used to determine the adjusted associations between CHE incidence and study area and between impoverishment status and study area. In these models, the education and occupation of the HH head, the HH size, healthcare utilization for chronic illness, healthcare utilization from private facility and UzHC accessibility were used as the covariates for adjustment.

The binary logistic regression models were specified as follows:


(2) $$Logit{\ }({Y_{ki}}) = {\theta _0} + {\theta _1}{X_{1i}} + {\theta _2}{X_{2i}} + {\theta _3}{X_{3i}} + \ldots + {u_i}$$

where *Y_k_* is the dependent variable of interest, e.g. CHE at 10% threshold or 40% threshold levels or impoverishment and coded as binary (0= No 1 = Yes), θ_0_ is a constant, *X*_1_ indicates study area with values 0 or 1 (0 = Comparison, 1 = Intervention), θ_1_ is the coefficient that shows the magnitude and direction of relationship, *X*_2_, *X*_3_, … denote control variables, θ_2_, θ_3_, … represent the estimated coefficients and *u_i_* is the random error term of the model.

A set of similar linear regression and binary logistic regression models were applied to assess the association of HHs’ BPL status and SSK service use status, separately, with the OOPE, CHE incidence and impoverishment, where the HHs’ BPL status/SSK service use status were the main independent variables.

## Results

In the intervention areas, approximately 58% (*n* = 4177) of the 7158 insured HHs were currently BPL HHs and 42% (*n* = 2981) were non-BPL HHs, as verified by the study team using the same BPL identification criteria as used by the SSK scheme. In the intervention areas, 16% (*n* = 1170) of the insured HHs (54% BPL and 46% non-BPL) had at least one incident of IPC in 12 months prior to the date of the interview. The corresponding figure in the comparison area was 15% (*n* = 1145). Among the insured HHs that sought IPC in the intervention area, only one-third (*n* = 372) utilized the SSK services. Of the remaining two-thirds, 45% (*n* = 798) sought healthcare from private providers, and 55% sought healthcare from public providers not designated as providing SSK services.

### Household characteristics

The similarities and differences in the characteristics of the BPL HHs in the comparison areas and each group of BPL and non-BPL HHs in the intervention areas are depicted in Table 1. Comparing the BPL HHs in the intervention and comparison areas, no variation was observed in terms of sex, education of the HH heads or IPC experience due to chronic illness between BPL HHs in the intervention and comparison areas. The proportion of three major occupations, e.g. daily labourer, rickshaw/auto driver and unemployed, were also similar between BPL HH in the intervention and comparison areas. However, the age of the HH heads and the HH size came out statistically significantly different between the intervention and comparison areas despite having a similar distribution pattern.

**Table 1.** Characteristics of study population

	Intervention areas		Comparison areas	
	BPL (*n* = 630)	Non-BPL (*n* = 540)	BPL (*n* = 1145)	
	Percentage (95% CI)	Percentage (95% CI)	Percentage (95% CI)	
Characteristics	A	B	C	*P*-value^a^
Age of the HH head				
Up to 30 years	10.5 (8.3–13.1)	8.5 (6.4–11.2)	17.4 (15.3–19.7)	<0.000^┴^
31–40 years	25.6 (22.3–29.1)	22.0 (18.7–25.7)	27.2 (24.7–29.8)	<0.000^◊^
41–50 years	26.3 (23.0–29.9)	21.1 (17.9–24.8)	23.1 (20.7–25.6)	
51–60 years	17.8 (15.0–21.0)	24.4 (21.0–28.3)	18.3 (16.1–20.6)	
60+	19.8 (16.9–23.1)	23.9 (20.5–27.7)	14.1 (12.2–16.3)	
Sex of HH head				
Male	90.3 (87.7–92.4)	88.1 (85.1–90.6)	89.3 (87.3–90.9)	<0.483^┴^
Female	9.7 (7.6–12.3)	11.9 (9.4–14.9)	10.7 (9.1–12.7)	<0.499^◊^
Education of HH head				
No institutional education	61.7 (57.9–65.5)	47.2 (43.0–51.5)	63.4 (60.6–66.2)	<0.690^┴^
Up to primary	24.1 (20.9–27.6)	24.1 (20.6–27.9)	21.7 (19.4–24.1)	<0.000^◊^
Secondary	12.7 (10.3–15.5)	23.7 (20.3–27.5)	13.4 (11.5–15.5)	
Higher secondary	1.4 (0.7–2.7)	5.0 (3.4–7.2)	1.6 (1.0–2.5)	
Occupation of the HH head				
Farmer	6.0 (4.4–8.2)	29.4 (25.7–33.4)	5.6 (4.4–7.1)	<0.015^┴^
Housewife	5.7 (4.1–7.8)	10.4 (8.1–13.2)	7.4 (6.0–9.1)	<0.000^◊^
Rickshaw/auto driver	8.3 (6.3–10.7)	7.2 (5.3–9.7)	12.0 (10.2–14.0)	
Small business	5.7 (4.1–7.8)	23.7 (20.3–27.5)	6.1 (4.9–7.7)	
Day labourer	62.2 (58.4–65.9)	10.4 (8.1–13.2)	56.7 (53.8–59.5)	
Unemployed	9.8 (7.7–12.4)	10.9 (8.6–13.9)	11.4 (9.6–13.3)	
Other (e.g. religious leader)	2.2 (1.3–3.7)	8.0 (6.0–10.6)	0.9 (0.5–1.6)	
HH size				
Less or equal to 3 persons	28.9 (25.5–32.6)	22.4 (19.1–26.1)	35.5 (32.7–38.3)	<0.002^┴^
4–5 persons	51.7 (47.8–55.6)	48.7 (44.5–52.9)	50.3 (47.4–53.2)	<0.000^◊^
6 persons or more	19.4 (16.5–22.6)	28.9 (25.2–32.9)	14.2 (12.3–16.4)	
At least one member sought care for chronic illness in the last 90 days				
No	75.2 (71.7–78.5)	61.9 (57.7–65.9)	74.7 (72.1–77.1)	<0.792^┴^
Yes	24.8 (21.5–28.3)	38.1 (34.1–42.3)	25.3 (22.9–27.9)	<0.000^◊^
At least one HH member utilized a private facility in the last 12 months				
No	70.0 (66.3–73.5)	62.2 (58.0–66.2)	53.7 (50.8–56.6)	<0.000^┴^
Yes	30.0 (26.5–33.7)	37.8 (33.8–42.0)	46.3 (43.4–49.2)	<0.000^◊^
Accessibility to Upazila health facility				
Easy	34.1 (30.5–37.9)	23.3 (19.9–27.1)	41.5 (38.7–44.4)	<0.000^┴^
Medium	42.7 (38.9–46.6)	47.2 (43.0–51.5)	26.4 (23.9–29.0)	<0.000^◊^
Difficult	23.2 (20.0–26.6)	29.4 (25.7–33.4)	32.1 (29.5–34.9)	

a Chi square test;

┴ difference between BPL in intervention and comparison areas (A vs C);

◊ difference between non-BPL in intervention and BPL in comparison areas (B vs C).

Variation was also noticed in the utilization of IPC in private facilities between BPL HHs in the intervention areas and the comparison (30% vs 46%, respectively). This may have been related to the use of the SSK scheme in the intervention areas. Accessibility to UzHCs also varied between these two groups, which might have resulted in variation in the number of villages that could not be captured due to incorrect information concerning the villages and BPL HHs, as well as the start of coronavirus 2019 pandemic. Finally, the characteristics of the non-BPL HHs in the intervention areas differed greatly from those of the BPL HHs in the comparison areas.

### Out-of-pocket expenditure for healthcare

The association between SSK membership and OOPE for healthcare by the BPL status of HHs and the use of SSK services in both the intervention and comparison areas is shown in Table 2. On average, the total annual HH OOPE for healthcare in the intervention areas was BDT 23 366 (USD 275.1; USD 1 = BDT 84.95)^1^ with a median of BDT 15 675, which was significantly lower (*P* < 0.001) than the HH OOPE in the comparison areas (BDT 24 757; USD 291.4) with a median of BDT 19 000 (not shown in table). The OOPE for medicine was the highest in both the intervention and comparison areas, followed by that for diagnostic tests. Upon comparing the OOPE by current BPL status in the intervention areas, we found that the BPL HHs had incurred significantly lower OOPE (BDT 19 314) than the non-BPL HHs (BDT 28 093) in the intervention areas and the BPL HHs in the comparison areas (BDT 24 757). When comparing the OOPE by SSK services utilization, we found that insured HHs in which at least one member utilized SSK services had statistically significantly lower OOPE (BDT 11 915) as compared with the insured HHs that did not utilize SSK services (BDT 28 704) in the intervention areas and the BPL HHs without SSK coverage in the comparison areas (BDT 24 757). Both the BPL HHs and the HHs that had used the SSK services at least once in the last 12 months had the lowest medicine expenditure (BDT 8741 and BDT 5667, respectively) and diagnostic test expenditure (BDT 2847 and BDT 1825, respectively) in the intervention areas.

**Table 2.** Out-of-pocket expenditure (OOPE) per household by below-poverty-line (BPL) status and utilization of Shasthyo Surokhsha Karmasuchi (SSK) cards by study areas

	Intervention areas					Comparison areas
	By BPL status of HHs		By utilization of SSK benefits			
	BPL HHs	Non-BPL HHs	At least one HH member used SSK services	No HH member used SSK services	Overall intervention areas	BPL HHs
Expenditure components/indicators	A	B	C	D	E	F
	Mean (SE) in BDT	Mean (SE) in BDT	Mean (SE) in BDT	Mean (SE) in BDT	Mean (SE) in BDT	Mean (SE) in BDT
Medical items	***n* = 630**	***n* = 540**	***n* = 372**	***n* = 798**	***n* = 1170**	***n* = 1145**
Consultation	740 (58)	951 (66)	493 (63)	998 (56)	837 (44)	772 (39)
Registration fee	38 (6)	50 (9)	16 (6)	56 (7)	44 (5)	27 (4)
Medicine cost	8741 (860)	11 197 (697)	5667 (393)	11 836 (798)	9875 (565)	10 563 (421)
Diagnostic cost	2847 (223)	4847 (401)	1825 (252)	4677 (299)	3770 (222)	3474 (199)
Hospital bed rent	137 (26)	704 (172)	13 (7)	579 (118)	399 (81)	474 (94)
Package of services	2905 (422)	4174 (663)	1178 (316)	4569 (535)	3491 (381)	4529 (386)
Operation cost	974 (160)	2334 (341)	573 (236)	2081 (239)	1602 (180)	1738 (174)
Total medical costs	**16 383 (1120)**	**24 257 (1420)**	**9765 (783)**	**24 797 (1229)**	**20 017 (898)**	**21 577 (758)**
Non-medical items						
Food cost	373 (28)	516 (42)	291 (32)	508 (32)	439 (24)	504 (30)
Transport cost	1681 (92)	2143 (109)	1210 (82)	2214 (95)	1895 (71)	1671 (70)
Attendant cost	577 (37)	860 (48)	497 (45)	806 (38)	708 (30)	622 (31)
Other cost (e.g. tips)	298 (22)	317 (25)	152 (23)	379 (21)	307 (17)	384 (17)
Total non-medical costs	**2930 (136)**	**3836 (176)**	**2150 (137)**	**3907 (145)**	**3348 (110)**	**3180 (118)**
Total OOPE per HH per year	**19 314 (1194)**	**28 093 (1537)**	**11 915 (869)**	**28 704 (1314)**	**23 366 (965)**	**24 757 (832)**
*P-*value *^a^* of total OOPE	*P < *0.001(A vs B) *P < *0.001 (A vs F)		*P < *0.001(C vs D) *P < *0.001 (C vs F)		*P < *0.001(E vs F)	

a Wilcoxon rank sum test.

### Incidence of catastrophic health expenditure and impoverishment

Variations in the estimates of HH consumption expenditure, CHE incidence and impoverishment by BPL status and use of SSK services in the intervention and comparison areas are shown in Table 3. Overall, there is statistically significant difference at 1% level in average monthly HH expenditure between intervention areas (BDT 25 115) and comparison areas (BDT 17 614). In the intervention areas, the BPL HHs had a lower total monthly expenditure (BDT 20 898) as compared with the non-BPL HHs (BDT 30 041). The total monthly HH expenditure was also observed to be lower for the HHs that used SSK services (BDT 21 811) as compared with the HHs that did not use SSK services (BDT 26 656) in the intervention areas.

**Table 3.** Household food, non-food, total expenditure and catastrophic health expenditure (CHE) by below-poverty-line (BPL) status and study areas

	Intervention areas							Comparison areas	
	By BPL status of HHs			By utilization of SSK benefits					
	BPL HHs	Non-BPL HHs	*P*-value between A and B (A and F)	At least one member used SSK benefits	No member used SSK benefits	*P*-value between C and D (C and F)	Overall intervention areas	BPL HHs	*P*-value between E and F
	*n* = 630	*n* = 540		*n* = 372	*n* = 798		*n* = 1170	*n* = 1145	
Indicators per HH	A	B		C	D		E	F	
Mean expenditure in BDT (SE mean)			^a^			^a^			^a^
Monthly food expenditure	10 869 (1198)	14 142 (1223)	<0.05 (<0.08)	11 695 (1149)	12 698 (1139)	0.58 (<0.01)	12 379 (858)	8973 (453)	<0.01
Monthly non-food expenditure	10 025 (419)	15 899 (705)	<0.01 (<0.01)	10 115 (573)	13 958 (525)	<0.00 (<0.01)	10 789 (393)	8641 (190)	<0.01
Total monthly expenditure	20 893 (1308)	30 041 (1426)	<0.01 (<0.01)	21 811 (1308)	26 656 (1286)	<0.02 (<0.01)	25 115 (973)	17 614 (511)	<0.01
Percentage (95% CI)			^b^			^b^			^b^
10% of total expenditure as threshold level	35.9 (32.2–39.7)	37.0 (33.1–41.2)	0.68 (<0.01)	19.1 (15.4–23.4)	44.5 (41.1–48.0)	<0.00 (<0.01)	36.4 (33.7–39.2)	54.6 (51.7–57.5)	<0.01
25% of total expenditure as threshold level	9.1 (6.9–11.8)	8.3 (6.3–10.7)	0.61 (<0.01)	5.9 (3.9–8.8)	9.9 (8.0–12.2)	0.02 (<0.01)	8.6 (7.2–10.4)	14.7 (12.7–16.8)	<0.01
30% of non-food expenditure as threshold level	22.8 (19.4–26.5)	23.8 (20.6–27.3)	0.67 (<0.01)	14.5 (11.3–18.5)	27.4 (24.5–30.6)	<0.00 (<0.01)	23.3 (21.0–25.8)	39.0 (36.2–41.8)	<0.01
40% of non-food expenditure threshold level	15.2 (12.6–18.3)	13.9 (11.2–17.1)	0.51 (<0.01)	10.2 (7.5–13.7)	16.7 (14.2–19.4)	<0.00 (<0.01)	14.6 (12.7–16.8)	25.6 (23.1–28.2)	<0.01
HHs that fall below PL before OOPE for healthcare (A)	17.8 (15.0–21.0)	6.3 (4.5–8.7)	<0.00 (0.33)	16.9 (13.4–21.1)	10.4 (8.5–12.7)	<0.00 (0.24)	12.5 (10.7–14.5)	19.7 (17.4–22.1)	<0.01
HHs that fall below PL after OOPE for healthcare (B)	25.4 (22.1–28.9)	11.9 (9.4–14.9)	<0.00 (<0.02)	22.6 (18.6–27.1)	17.5 (15.1–20.3)	<0.04 (<0.01)	19.1 (17.0–21.5)	30.5 (27.9–33.2)	<0.01
HHs that fall below PL due to OOPE for healthcare (B—A)	7.6 (5.8–10.0)	5.6 (3.9–7.8)	0.15 (<0.02)	5.6 (3.7–8.5)	7.1 (5.5–9.2)	0.33 (<0.01)	6.7 (5.4–8.2)	10.8 (9.2–12.8)	<0.01

The estimated poverty line for the studied BPL population was BDT 2101/per capita or BDT 9038/ HH/month, which was similar to the national poverty line for the Dhaka rural area as estimated in the HH Income and Expenditure Survey 2016 (9305 BDT/HH/month or 2152 per capita/month) (Bangadesh Bureau of Statistics, 2017).

a t-test;

b z-test of proportion; PL: estimated poverty line 9038 BDT/HH/month or 2101 per capita/month.

**Figure 3.**
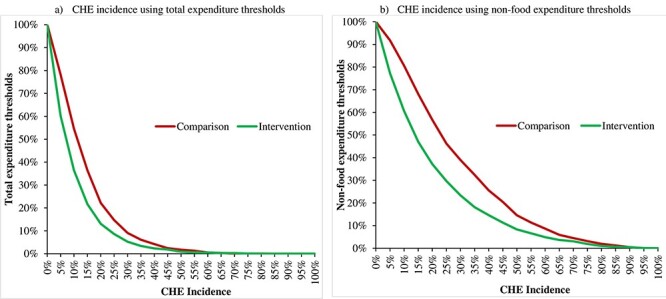
HHs’ CHE incidence by study areas considering different thresholds

Overall, depending on the threshold used for measuring CHE incidence, the CHE incidence ranged from 8.6% to 36.4% in the intervention areas and from 14.7% to 54.6% in the comparison areas. The CHE incidence in the intervention areas compared with that in the comparison areas was significantly lower such as at 10% threshold levels (36.4% vs 54.6%, respectively; *P* < 0.01) and at 40% threshold levels (14.6% vs 25.6%, respectively; *P* < 0.01). Concerning the BPL status of the HHs in the intervention areas, the incidence of CHE among the BPL HHs was significantly (*P* < 0.01) lower by 18.7% and 13.4% than the corresponding figures in the comparison areas (at 10% threshold: 35.9% vs 54.6%, respectively, and at 40% threshold: 12.2% vs 25.6%, respectively). The HHs that utilized SSK services had a significantly lower CHE incidence at 1% level considering both threshold levels in the intervention areas compared with those in the comparison areas (at 10% threshold: 19.1% vs 54.6%, respectively, and at 40% threshold: 10.2% vs 25.6%, respectively). CHE incidence sensitivity analyses showed that CHE incidence for both intervention and comparison area was higher when using a non-food expenditure threshold (Figure 3).

Overall, impoverishment from OOPE was significantly lower (*P* <0.01) in the intervention areas than in the comparison areas (6.7% vs 10.8%, respectively). In the intervention areas, BPL HHs (7.6% vs 10.8%, respectively; *P* < 0.01) and HHs that used SSK services (5.6% vs 10.8%, respectively; *P* < 0.01) experienced a significantly lower level of impoverishment due to OOPE than BPL HHs in the comparison areas. However, in the intervention areas, the BPL HHs experienced a relatively higher level of impoverishment due to OOPE than the non-BPL HHs (7.6% vs 5.6%, respectively; *P* = 0.15), although the difference was not statistically significant.

### Factors associated with the out-of-pocket expenditure for healthcare

The results of the linear regression analysis on the effect of the SSK services on HH OOPE for healthcare by overall study area, HH BPL status and HH utilization of SSK services are shown in Table 4. After adjustment, we found that the overall OOPE was significantly lower (*P* < 0.05), by 33%, among the insured HHs compared with the BPL HHs in the comparison areas. Considering HH BPL status as the main independent variable, OOPE was lower in both the non-BPL and BPL HHs by 21% and 40%, respectively, in the intervention areas compared with the BPL HHs in the comparison areas. Considering the status of SSK service utilization as a main independent variable, OOPE was 92% lower (*P* < 0.01) among the HHs that utilized SSK services compared with those that did not utilize SSK services in the comparison areas (Table 4).

**Table 4.** Association of natural log of out-of-pocket expenditure (OOPE) with Shasthyo Surokhsha Karmasuchi (SSK) membership status, below-poverty-line (BPL) status and SSK benefits used status while adjusting for other covariates

			Main independent variable = Overall membership	Main independent variable = BPL status	Main independent variable = SSK benefits use status
Characteristics	*n*	Mean (SE mean)	Adjusted coef. (95% CI)	Adjusted coef. (95% CI)	Adjusted coef. (95% CI)
Study area					
Comparison	1145	24 757 (832)	Ref.		
Intervention	1170	23 366 (965)	–0.33^b^ (–0.41,–0.24)		
BPL status of the HH					
BPL in comparison	1145	24 757 (832)		Ref.	
Non-BPL in intervention	540	19 314 (1194)		–0.21^b^ (–0.33,–0.09)	
BPL in intervention	630	28 093 (1537)		–0.40^b^ (–0.49,–0.30)	
Status of using SSK benefits					
Did not use SSK benefits (comparison)	1145	24 757 (832)			Ref.
Did not use SSK benefits (intervention)	798	28 704 (1314)			–0.05 (–0.14,0.04)
Used SSK benefits (intervention)	372	11 915 (869)			–0.92^b^ (–1.08,–0.76)
At least one member sought care for chronic illness in last 90 days					
No	1663	20 990 (698)	Ref.	Ref.	Ref.
Yes	652	31 870 (1355)	0.63^b^ (0.53,0.74)	0.62^b^ (0.52,0.73)	0.62^b^ (0.52,0.72)
At least one member utilized private facility in last 12 months					
No	1392	18 000 (775)	Ref.	Ref.	Ref.
Yes	923	33 183 (1024)	0.95^b^ (0.85,1.05)	0.95^b^ (0.85,1.04)	0.81^b^ (0.72,0.90)
Accessibility to UzHCs					
Easy	816	22 523 (1018)	Ref.	Ref.	Ref.
Medium	826	24 910 (1200)	0.12^a^ (0.03,0.21)	0.11^a^ (0.02,0.20)	0.06 (–0.02,0.14)
Difficult	673	24 860 (1061)	0.09 (–0.02,0.20)	0.08 (–0.03,0.19)	0.02 (–0.10,0.14)
Education of HH head					
No institutional education	1370	22 639 (756)	Ref.	Ref.	Ref.
Up to primary	530	23 193 (1007)	0.07 (–0.04,0.17)	0.06 (–0.04,0.17)	0.06 (–0.04,0.15)
Secondary	361	30 290 (2438)	0.32^b^ (0.22,0.42)	0.31^b^ (0.20,0.41)	0.28^b^ (0.18,0.38)
Higher secondary	54	26 697 (3567)	0.36^b^ (0.10,0.62)	0.34^a^ (0.08,0.60)	0.29^a^ (0.07,0.51)
Occupation of the HH head					
Agriculture	261	26 913 (1578)	Ref.	Ref.	Ref.
Housewife	177	25 042 (2548)	–0.09 (–0.31,0.13)	–0.06 (–0.28,0.17)	–0.09 (–0.30,0.11)
Rickshaw/auto driver	228	23 227 (1616)	–0.09 (–0.24,0.05)	–0.04 (–0.19,0.12)	–0.05 (–0.20,0.11)
Small business	234	32 794 (3466)	0.07 (–0.08,0.21)	0.08 (–0.06,0.21)	0.06 (–0.08,0.20)
Day labourer/worker	1097	20 818 (701)	–0.17^b^ (–0.30,–0.05)	–0.10 (–0.23,0.04)	–0.15^a^ (–0.27,–0.04)
Unemployed	251	27 648 (2406)	–0.11 (–0.30,0.08)	–0.07 (–0.26,0.13)	–0.06 (–0.26,0.13)
Other	67	22 110 (2704)	–0.17 (–0.44,0.11)	–0.16 (–0.44,0.11)	–0.16 (–0.41,0.09)
HH size (equivalence scale)					
Less or equal to 3 persons	709	22 510 (1083)	Ref.	Ref.	Ref.
4–5 persons	1165	23 385 (841)	0.14^b^ (0.04,0.23)	0.13^b^ (0.04,0.22)	0.11^a^ (0.01,0.20)
6 persons or more	441	28 302 (1795	0.21^b^ (0.09,0.33)	0.19^b^ (0.07,0.31)	0.17^b^ (0.05,0.29)

a *P* < 0.05;

b *P* < 0.01.

### Factors associated with the incidence of catastrophic healthcare expenditure

The logistic regression analysis showed that, after adjustment for covariates, the CHE incidence in the intervention areas remained significantly lower (Odds ratio (OR): 0.54; *P* < 0.01) than in the comparison areas (Table 5) at 10% of total HH expenditure as a threshold. Both non-BPL HHs and BPL HHs in the intervention areas had a significantly lower CHE incidence compared with the BPL HHs in the comparison areas. However, the OR was lower for the non-BPL HHs in the intervention areas compared with the BPL HHs in the intervention areas (OR: 0.48 vs 0.57, respectively). HHs in the intervention areas that utilized SSK services were less likely (OR: 0.28; *P* < 0.01) to experience CHE compared with the HHs in the comparison areas. Even when SSK service utilization was considered, the non-BPL HHs in the intervention areas were significantly less likely to experience CHE compared with the HHs in the comparison areas. Overall, HHs that utilized healthcare for chronic illness were more than twice as likely to experience CHE (OR: 2.57; *P* < 0.01) and the HHs that utilized healthcare from private facilities were about four times (OR: 4.46; *P* < 0.01) as likely to face CHE compared with the HHs those did not utilize either of these services. The CHE findings were si–milar at the 40% threshold level.

**Table 5.** Association of incidence of catastrophic health expenditure (CHE) (at 10% of total expenditure and 40% of non-food expenditure) with Shasthyo Surokhsha Karmasuchi (SSK) membership, below-poverty-line (BPL) status and SSK benefits use status while adjusting for other covariates

	Dependent variable = CHE at 10% threshold				Dependent variable = CHE at 40% threshold				
	Per cent of CHE incidence (95% CI)	Main independent variable = Overall membership	Main independent variable = BPL status	Main independent variable = SSK benefits use status	Percentage of CHE incidence (95%CI)	Crude OR (95% CI)	Main independent variable = Overall membership	Main independent variable = BPL status	Main independent variable = SSK benefits use status
Characteristics		Adjusted OR (95% CI)	Adjusted OR (95% CI)	Adjusted OR (95% CI)			Adjusted OR (95% CI)	Adjusted OR (95% CI)	Adjusted OR (95% CI)
Study area									
Comparison	54.6 (51.7–57.5)	Ref.			25.6 (23.1–28.2)	Ref.	Ref.		
Intervention	36.4 (33.7–39.2)	0.54^c^ (0.44,0.66)			14.6 (12.7–16.8)	0.50^c^ (0.40,0.61)	0.58^c^ (0.45,0.75)		
BPL status of the HH									
BPL in comparison	54.6 (51.7–57.5)		Ref.		25.6 (23.1–28.2)	Ref.		Ref.	
Non-BPL in intervention	37.0 (33.1–41.2)		0.48^c^ (0.36,0.65)		13.9 (11.2–17.1)	0.47^c^ (0.36,0.62)		0.50^c^ (0.34,0.74)	
BPL in intervention	35.9 (32.2–39.7)		0.57^c^ (0.46,0.72)		15.2 (12.6–18.3)	0.52^c^ (0.41,0.67)		0.63^c^ (0.48,0.84)	
Status of using SSK benefits									
Did not use SSK benefits (comparison)	54.6 (51.7–57.5)			Ref.	25.6 (23.1–28.2)	Ref.			Ref.
Did not use SSK benefits (intervention)	44.5 (41.1–48.0)			0.70^c^ (0.56,0.87)	16.7 (14.2–19.4)	0.58^c^ (0.46,0.73)			0.66^c^ (0.50,0.86)
Used SSK benefits (intervention)	19.1 (15.4–23.0)			0.28^c^ (0.20,0.40)	10.2 (7.5–13.7)	0.33^c^ (0.23,0.47)			0.41^c^ (0.28,0.60)
At least one member sought care for chronic illness in last 90 days									
No	40.6 (38.3–43.0)	Ref.	Ref.	Ref.	17.5 (15.7–19.4)	Ref.	Ref.	Ref.	Ref.
Yes	57.7 (53.8–61.4)	2.57^c^ (2.05,3.23)	2.60^c^ (2.07,3.27)	2.60^c^ (2.06,3.28)	26.5 (23.3–30.1)	1.70^c^ (1.37,2.11)	1.98^c^ (1.51, 2.60)	2.00^c^ (1.53,2.63)	1.98^c^ (1.51,2.60)
At least one member utilized private facility in last 12 months									
No	32.8 (30.3–35.3)	Ref.	Ref.	Ref.	15.2 (13.4–17.1)	Ref.	Ref.	Ref.	Ref.
Yes	64.5 (61.3–67.5)	4.46^c^ (3.69,5.40)	4.48^c^ (3.70,5.42)	3.96^c^ (3.27,4.79)	27.4 (24.6–30.4)	2.11^c^ (1.72,2.60)	2.74^c^ (2.18,3.44)	2.76^c^ (2.20,3.47)	2.58^c^ (2.03,3.27)
Accessibility to UzHCs									
Easy	44.2 (40.9–47.7)	Ref.	Ref.	Ref.	19.1 (16.6–22.0)	Ref.	Ref.	Ref.	Ref.
Medium	42.3 (38.9–45.7)	1.07 (0.86,1.33)	1.08 (0.87,1.34)	1.02 (0.82,1.27)	19.5 (16.9–22.3)	1.02 (0.80,1.31)	1.27 (0.94,1.73)	1.28 (0.95,1.75)	1.25 (0.92,1.71)
Difficult	50.7 (46.9–54.4)	1.26^a^ (0.98,1.62)	1.27^a^ (0.99,1.64)	1.19 (0.92,1.53)	21.8 (18.9–25.1)	1.18 (0.92,1.52)	1.14 (0.82,1.59)	1.15 (0.82,1.60)	1.12 (0.80,1.55)
Education of HH head									
No institutional education	45.7 (43.1–48.3)	Ref.	Ref.	Ref.	21.3 (19.2–23.6)	Ref.	Ref.	Ref.	Ref.
Up to primary	43.0 (38.9–47.3)	0.9 (0.70,1.15)	0.9 (0.71,1.16)	0.88 (0.69,1.13)	18.1 (15.1–21.6)	0.82 (0.63,1.05)	0.9 (0.67,1.22)	0.91 (0.68,1.23)	0.89 (0.66,1.20)
Secondary	47.4 (42.3–52.5)	1.24 (0.96,1.59)	1.25^a^ (0.97,1.61)	1.19 (0.92,1.53)	19.1 (15.4–23.5)	0.87 (0.65,1.17)	1.1 (0.80,1.51)	1.12 (0.82,1.53)	1.08 (0.78,1.48)
Higher secondary	48.1 (35.1–61.4)	1.3 (0.71,2.39)	1.33 (0.72,2.45)	1.21 (0.68,2.15)	13.0 (6.3–24.9)	0.55 (0.25,1.23)	0.57 (0.22,1.51)	0.58 (0.22,1.54)	0.55 (0.21,1.48)
Occupation of the HH head									
Agriculture	41.8 (35.9–47.9)	Ref.	Ref.	Ref.	16.5 (12.4–21.5)	Ref.	Ref.	Ref.	Ref.
Housewife	51.4 (44.0–58.7)	1.29 (0.82,2.05)	1.26 (0.79,2.00)	1.31 (0.83,2.05)	35.0 (28.3–42.4)	2.73^c^ (1.74,4.29)	1.87^c^ (1.18,2.94)	1.81^b^ (1.15,2.85)	1.88^c^ (1.19,2.97)
Rickshaw/auto driver	40.4 (34.2–46.9)	0.83 (0.57,1.19)	0.78 (0.53,1.15)	0.87 (0.60,1.26)	8.3 (5.4–12.7)	0.46^c^ (0.26,0.82)	0.36^c^ (0.19,0.66)	0.34^c^ (0.18,0.63)	0.36^c^ (0.19,0.68)
Small business	43.2 (36.9–49.6)	1.05 (0.73,1.51)	1.04 (0.72,1.50)	1.06 (0.74,1.53)	12.0 (8.4–16.8)	0.69 (0.41,1.15)	0.66 (0.39,1.12)	0.65 (0.39,1.10)	0.67 (0.39,1.13)
Day labourer/worker	45.3 (42.4–48.3)	1.13 (0.83,1.54)	1.05 (0.76,1.44)	1.16 (0.85,1.57)	19.0 (16.7–21.4)	1.19 (0.83,1.70)	1.04 (0.71,1.52)	0.96 (0.65,1.41)	1.05 (0.72,1.54)
Unemployed	54.2 (48.0–60.3)	1.61^b^ (1.09,2.38)	1.55^b^ (1.02,2.34)	1.71^c^ (1.15,2.53)	37.5 (31.7–43.6)	3.04^c^ (2.00,4.60)	2.52^c^ (1.67,3.78)	2.39^c^ (1.59,3.60)	2.58^c^ (1.71,3.87)
Other	37.3 (26.5–49.5)	1.01 (0.52,1.98)	1.01 (0.52,1.97)	1.03 (0.53,2.02)	14.9 (8.2–25.7)	0.89 (0.42,1.88)	1.11 (0.47,2.59)	1.11 (0.48,2.59)	1.13 (0.48,2.66)
HH size (equivalence scale)									
Less or equal to 3 persons		Ref.	Ref.	Ref.	34.3 (30.9–37.9)	34.3 (30.9–37.9)	34.3 (30.9–37.9)	34.3 (30.9–37.9)	34.3 (30.9–37.9)
4–5 persons	43.8 (40.9–46.6)	0.59^c^ (0.48,0.73)	0.60^c^ (0.48,0.73)	0.57^c^ (0.46,0.70)	14.8 (12.9–17.0)	14.8 (12.9–17.0)	14.8 (12.9–17.0)	14.8 (12.9–17.0)	14.8 (12.9–17.0)
6 persons or more	34.5 (30.2–39.0)	0.33^c^ (0.25,0.43)	0.33^c^ (0.25,0.44)	0.31^c^ (0.24,0.41)	10.9 (8.3–14.2)	10.9 (8.3–14.2)	10.9 (8.3–14.2)	10.9 (8.3–14.2)	10.9 (8.3–14.2)

a *P* < 0.10;

b *P* < 0.05;

c *P* < 0.01.

### Factors associated with the incidence of impoverishment

The incidence of impoverishment among the HHs in the intervention areas was significantly lower (OR: 0.70; *P* < 0.01) compared with the HHs in the comparison areas **(**Table 6). While testing the association considering HH BPL status as the main independent variable, both non-BPL and BPL HHs were less likely to be impoverished compared with the BPL HHs in the comparison areas. However, the association was only significant for the non-BPL HHs in the intervention areas. When using SSK service usage as the main independent variable, the HHs in the intervention areas those used SSK benefits (OR: 0.73; *P* < 0.10) and the HHs those did not use SSK benefits were significantly less likely to be impoverished (OR: 0.69; *P* < 0.001) compared with the HHs in the comparison area. However, the association of lower incidence of impoverishment for HHs those used SSK benefits was significant at 10% level of significance.

**Table 6.** Association of impoverishment with Shasthyo Surokhsha Karmasuchi (SSK) membership status, below-poverty-line (BPL) status and SSK benefits use status while adjusting for other covariates

	Percentage of impoverishment(95% CI)	Main independent variable = Overall membership	Main independent variable = BPL status	Main independent variable = SSK benefits use status
Characteristics		Adjusted OR (95% CI)	Adjusted OR (95% CI)	Adjusted OR (95% CI)
Study area				
Comparison	30.5 (27.9–33.2)	Ref.		
Intervention	19.1 (17.0–21.5)	0.70^c^ (0.54,0.91)		
BPL status of the HH				
BPL in comparison	30.5 (27.9–33.2)		Ref.	
Non-BPL in intervention	11.9 (9.4–14.9)		0.44^c^ (0.29,0.66)	
BPL in intervention	25.4 (22.1–28.9)		0.86 (0.65,1.13)	
Status of using SSK benefits				
Did not use SSK benefits (comparison)	30.5 (27.9–33.2)			Ref.
Did not use SSK benefits (intervention)	17.5 (15.1–20.3)			0.69^c^ (0.52,0.91)
Used SSK benefits (intervention)	22.6 (18.6–27.1)			0.73^a^ (0.52,1.02)
At least one member sought care for chronic illness in last 90 days				
No	25.7 (23.6–27.8)	Ref.	Ref.	Ref.
Yes	22.4 (19.4–25.8)	0.81 (0.62,1.05)	0.83 (0.64,1.08)	0.81 (0.62,1.05)
At least one member utilized private facility in last 12 months				
No	26.0 (23.8–28.4)	Ref.	Ref.	Ref.
Yes	22.9 (20.3–25.7)	0.97 (0.79,1.19)	0.99 (0.81,1.22)	0.98 (0.79,1.21)
Accessibility to UzHCs	(-)			
Easy	24.1 (21.3–27.2)	Ref.	Ref.	Ref.
Medium	21.9 (19.2–24.9)	1.12 (0.82,1.53)	1.16 (0.85,1.58)	1.12 (0.82,1.53)
Difficult	29.0 (25.7–32.5)	1.37^b^ (1.02,1.85)	1.41^b^ (1.04,1.91)	1.38^b^ (1.02,1.85)
Education of HH head				
No institutional education	28.6 (26.3–31.1)	Ref.	Ref.	Ref.
Up to primary	20.0 (16.8–23.6)	0.66^c^ (0.50,0.87)	0.68^c^ (0.52,0.89)	0.66^c^ (0.50,0.87)
Secondary	18.0 (14.4–22.3)	0.64^c^ (0.47,0.88)	0.68^b^ (0.50,0.93)	0.65^c^ (0.47,0.88)
Higher secondary	18.5 (10.2–31.3)	0.67 (0.26,1.74)	0.71 (0.27,1.86)	0.68 (0.26,1.75)
Occupation of the HH head				
Agriculture	13.8 (10.1–18.5)	Ref.	Ref.	Ref.
Housewife	44.6 (37.5–52.0)	2.86^c^ (1.76,4.64)	2.56^c^ (1.56,4.19)	2.86^c^ (1.76,4.63)
Rickshaw/auto driver	9.2 (6.1–13.7)	0.49^b^ (0.25,0.93)	0.40^c^ (0.21,0.79)	0.49^b^ (0.25,0.93)
Small business	10.3 (7.0–14.9)	0.77 (0.45,1.33)	0.74 (0.42,1.30)	0.77 (0.45,1.33)
Day labourer/worker	26.4 (23.9–29.1)	1.93^c^ (1.32,2.82)	1.49^a^ (0.97,2.29)	1.92^c^ (1.32,2.81)
Unemployed	45.0 (39.0–51.2)	4.12^c^ (2.65,6.40)	3.53^c^ (2.18,5.70)	4.11^c^ (2.64,6.39)
Other	14.9 (8.2–5.7)	1.21 (0.51,2.89)	1.17 (0.49,2.80)	1.2 (0.50,2.88)
HH size (equivalence scale)				
Less or equal to 3 persons	50.2 (46.5–53.9)	Ref.	Ref.	Ref.
4–5 persons	16.7 (14.7–19.0)	0.22^c^ (0.18,0.28)	0.23^c^ (0.18,0.29)	0.22^c^ (0.18,0.28)
6 persons or more	5.0 (3.3–7.5)	0.06^c^ (0.04,0.09)	0.06^c^ (0.04,0.09)	0.06^c^ (0.04,0.09)

a *P* < 0.10;

b *P* < 0.05;

c *P* < 0.01.

## Discussion

Overall, the SSK scheme significantly reduced the OOPE and the CHE incidence among the HHs (both BPL and non-BPL) in the intervention areas compared with those in the comparison areas. The scheme also had an overall significant effect on protecting the HHs from impoverishment. However, considering the association of HH BPL status with impoverishment, only the non-BPL HHs were less likely to be impoverished in the intervention area compared with the BPL HHs in the comparison area. This comparative study was the first attempt to evaluate the effect of the pilot SSK health protection scheme on OOPE, CHE and economic impoverishment among the enrolled HHs.

Significant associations of OOPE and CHE with two other variables, i.e. HHs’ current BPL status and SSK benefit utilization, were identified. The OOPE among the BPL HHs in the intervention areas was significantly lower compared with the BPL HHs in the comparison areas and non-BPL HHs in the intervention areas. This might have been because the non-BPL HHs belonged to a higher socioeconomic group with a higher capacity to pay for healthcare services. Thus, these HHs might have utilized more healthcare from private facilities, ultimately increasing their overall OOPE. Furthermore, upon comparing compliant HHs (those HHs that used SSK benefits) in the intervention areas and HHs in the comparison areas, the OOPE was significantly lower among the former group.

The literature in Bangladesh on the effect of health insurance schemes on OOPE has reported mixed outcomes. For example, a quasi-experimental study examined the effect of a community-based health insurance scheme on OOPE among enrolees and found that OOPE was 6.4% lower among the insured HHs that utilized medically trained providers (Khan *et al.*, 2020). An earlier study on the effect of a compulsory employer-sponsored health insurance scheme among ready-made garment workers in Bangladesh found that although the scheme increased the utilization of healthcare from formal healthcare providers, it had no significant effect on the reduction of OOPE for healthcare among the insured HHs (Ahmed *et al.*, 2020). Similarly, at the international level, several studies have shown significant positive effects of insurance in reducing OOPE (Fan *et al.*, 2012; Harish *et al.*, 2020), while others have demonstrated non-significant or negative effects (Dror *et al.*, 2016). A study on the impact of a community-based health insurance scheme in Burkina Faso found that the scheme had a limited effect on average OOPE for healthcare (Fink *et al.*, 2013). Concerning publicly financed health insurance schemes in India, research has revealed that beneficiaries of the insurance schemes incurred OOPE during hospitalization (Devadasan *et al.*, 2013) and that the scheme had no effects in reducing burden of OOPE among poor beneficiaries (Karan *et al.*, 2017). It was found that medicine expenditure was the highest among the components of OOPE for healthcare. Similar findings have been reported in previous studies conducted in local and global contexts. For instance, the Bangladesh National Health Accounts for 2015 showed that medicine expenditure was the highest driver of OOPE in Bangladesh (MOHFW, 2015). Moreover, Mahumud *et al.*, (2017) found that 61% of total OOPE for healthcare was driven by medicine costs in Bangladesh. Shahrawat and Rao (2012) calculated that medicine costs constituted approximately 72% of total OOPE in India.

The current study determined that, overall, the SSK scheme significantly reduced the CHE incidence in the intervention areas compared with the comparison areas at all threshold levels used. The CHE incidence was significantly lower among the HHs that used SSK benefits as compared with the overall intervention and comparison HHs. Moreover, the CHE incidence was lower among the non-BPL HHs in the intervention areas. This may have been because the non-BPL HHs had a higher income as compared with the BPL HHs in both intervention and comparison areas.

Several of the insured HHs in the intervention areas did not fulfil the BPL selection criterion (identified as non-BPL) during the HH screening process by the study team. It is unknown whether the SSK program identified BPL HHs had graduated from BPL to non-BPL status or there were errors in the HHs selection during SSK scheme enrolment. It is likely that the CHE incidence was more concentrated among the poorer HHs compared with the richer ones (Khan *et al.*, 2017). The HHs that did not use SSK benefits in the intervention areas had a lower CHE incidence compared with the comparison areas. Such non-user HHs included both poor BPL and rich non-BPL HHs (did not fulfil the BPL selection criteria during the HH selection for this study) in the intervention areas. The rich HHs were less likely to face CHE. Thus, overall, HHs that did not use SSK benefits in the intervention areas also had a lower incidence of CHE vs the HHs in the comparison areas.

Regarding the effect of health insurance schemes on CHE, similar findings were observed in the literature. Aryeetey *et al.*, (2016) conducted a study in 2016 on Ghana’s National Health Insurance Scheme and found that the CHE incidence was lower among the insured HHs (7–18%) compared with the uninsured HHs (29–36%). Fink *et al.*, (2013) evaluated the welfare and health impact of Burkina Faso’s insurance program in 2013 and found a lower CHE incidence among the poor and vulnerable groups. Xu *et al.*, (2018) evaluated the impact of the Chinese government’s New Health Care Reform scheme and discovered that it significantly reduced CHE among beneficiaries. Tirgil *et al.*, (2019) evaluated the effects of the Green Card scheme on OOPE for low-income HHs in Turkey in 2019 and determined that it reduced CHE incidence by 50% among the vulnerable HHs with largest annual OOPE.

The association of impoverishment with the study areas showed that overall impoverishment was significantly lower by 30% in the intervention areas compared with the comparison areas. The scheme protected more than 4% of the HHs from being pushed into poverty due to OOPE for healthcare as compared with the comparison area. When the association was tested using HHs’ BPL status as a main independent variable, the non-BPL HHs in the intervention areas were less likely to become impoverished compared with the BPL HHs in the comparison areas. This might have been because the non-BPL HHs belonged to the higher-income group and had higher capacity to pay compared with the BPL HHs in both areas (1.5 and 1.7 times higher than the BPL HHs in intervention and comparison areas, respectively).

There is mixed evidence on the effect of a health protection scheme on HH impoverishment. For instance, a study conducted on Ghana’s National Health Insurance Scheme in 2016 reported that the scheme protected 7.5% points of the enrolled HHs in the vulnerable group from being pushed into poverty (Aryeetey *et al.*, 2016). However, an evaluation of the Aarogyasri health insurance scheme among the BPL population in India indicated that the scheme had no clear effects on economic impoverishment (Fan *et al.*, 2012).

This study found that an HH was more likely to face CHE if any HH member had utilized private facilities within the 12 months before the survey. The association of impoverishment with seeking care from private healthcare facilities was significant at the 10% level of significance. This finding was similar to a previous study conducted in Bangladesh that found that the CHE incidence was 9.88 times higher if at least one member utilized healthcare from private providers (Ahmed *et al.*, 2022). Treatment costs at a private facility are high which increases the risk of facing CHE (Hasan *et al.*, 2021). The current study found that HHs in areas in which it is difficult to access a healthcare facility incurred a significantly higher incidence of CHE compared with HHs in areas with easy access. This might be because lack of easy access to healthcare facilities is associated with increased non-medical costs, such as for transport, food and accommodation. A 2016 study conducted among a poor population in Zambia showed that cost due to distance is associated with a higher likelihood of experiencing CHE (Masiye *et al.*, 2016). A recent study in Kenya reported that CHE incidence and impoverishment are associated with the distance travelled to seek healthcare (Njuguna *et al.*, 2017).

The current study identified that 18 of the 90 selected villages had no SSK BPL population and that 46% of the surveyed HHs in the intervention areas did not comply with the BPL selection criterion. The enrolment of HHs was supposed to follow a house-to-house visit to identify BPL HHs by verifying the BPL selection criteria. This process was not properly followed by the scheme operator. Moreover, political and local power structures might have influenced the accuracy of the SSK BPL list. Thus, before future expansion of the SSK program, it is suggested that a clear strategy be developed and implemented to identify BPL HHs by checking the BPL selection criteria through door-to-door visits. A mechanism should be developed to regularly update the list of insured BPL HHs.

Despite being insured by the SSK scheme, about one-third of the HHs utilized SSK benefits and incurred lower OOPE, CHE incidence and impoverishment. The utilization of SSK scheme is not high. An earlier study conducted by Hasan *et al.*, (2022) showed similar findings of low utilization of SSK scheme and identified that utilization was linked to the knowledge about the scheme and its benefits package. The issue of low utilization should be investigated, and necessary initiative should be taken to improve utilization of SSK scheme among the insured HHs.

Moreover, the SSK scheme covered healthcare expenditure for 78 disease groups. This may limit the utilization of the scheme by excluding patients who needed care for conditions outside of this range. Thus, to improve the utilization of the scheme, a comprehensive benefits package should be developed based on the healthcare needs of the BPL population.

SSK is a non-contributory health protection scheme which is financed through government general revenues for services without any contribution from the enrolled HHs. The healthcare financing strategy of the government of Bangladesh outlined that the formal sector employees, including government and private sector workers, will be covered by SSK with payroll taxes and employer contributions. However, it is important to note that the informal segment of population have irregular income and are geographically dispersed. It will be difficult to reach this group with a formal financing mechanism in short term and it is anticipated that their enrolment with voluntary subscription to SSK will be very low. Integration of contributory feature by payroll tax, employer contribution or voluntary subscriptions into the social health insurance scheme is not proven to be effective (Yazbeck *et al.*, 2020). Evidence showed that when a country’s health financing policy shifts from reliance on OOPE for healthcare to social health insurance schemes, non-contributory schemes are more successful in improving health system outcomes compared with the contributory schemes (Yazbeck *et al.*, 2020; Gabani *et al.*, 2023). Thus, the inclusion of both formal sector and informal sector under the SSK scheme through pay roll tax/employer funding and voluntary subscriptions, respectively, should be decided carefully and supported by evidence in the context of Bangladesh. Given the effectiveness of the SSK scheme in reducing the financial burden on the BPL HHs, the government can gradually scale up the non-contributory scheme. This phased approach can help to cope up with the rising cost of expansion and reduce pressure on the general revenues. Moreover, the government may consider increasing the general tax and financial contributions to the SSK scheme from it to cover the increased costs associated with scaling up the non-contributory scheme. However, this is albeit a long-term process (Yazbeck *et al.*, 2023).

### Limitations of the study

One possible limitation of this research is that it was conducted as a cross-sectional study with a comparison group. A longitudinal study with a comparison group might have captured the true effect of the SSK scheme on the enrolled HHs compared with the HHs in the comparison areas over the intervention period. However, this is the first study to evaluate the effects of the SSK scheme on the key financial indicators of enrolled BPL HHs in Bangladesh. Future research could use the information obtained here to compare the effect of the scheme to track its performance towards its goal. Another limitation concerns the fact that HHs in which one member had received IPC in the last 12 months were surveyed to obtain information on the history of illness, healthcare utilization and OOPE, with a recall period of 12 months for IPC and 3 months for outpatient care. It is possible that recall bias and self-reported information bias could have occurred in the respondents’ recounting of the history of illness, healthcare utilization and OOPE for healthcare due to poor knowledge of medical conditions and healthcare services. However, since IPC is a major event for a patient, capturing information for the last 12 months may have minimized such biases. Furthermore, many of the HHs might have foregone care before their enrolment in the scheme (did not seek healthcare even when the individual perceived it was required). The policy might have affected the foregone care of the insured HHs which we could not capture. The measures of financial risk protection like CHE and impoverishment do not account for forgone care; for this reason, we could not consider such care in this study (Moreno-Serra *et al.*, 2011). Finally, we could not capture the effect of the SSK scheme on the patients who were not covered through this scheme (uninsured). As the public hospitals are main healthcare provider of the SSK insured patients and utilizes medicine and investigation from regular supplies, there are potential of negatives effects on the uninsured patients who utilize healthcare from these facilities.

## Conclusion

The findings of this study demonstrate that in Bangladesh, the use of government-funded health protection scheme among the BPL population could reduce the overall OOPE for healthcare. Furthermore, it could reduce the CHE incidence and protect HHs from impoverishment. This necessitates the importance of community engagement and motivational campaigns in increasing the utilization of the health protection scheme among the enrolled BPL HHs. The evidence also suggests that policymakers should take steps to reduce the selection bias while identifying BPL HHs for the effective use of resources who need it the most. These findings may aid in decision-making for the necessary refinement of the SSK scheme before it is scaled up in other areas to accelerate the timely achievement of the universal health coverage and sustainable development goal targets.

## Supplementary Material

czad115_Supp

## Data Availability

The data underlying this article will be shared on reasonable request to the corresponding author.

## Footnotes

1. Exchange rate of United States Dollar (USD) to Bangladeshi Taka (BDT), March 2020, Bangladesh Bank.

## Reflexivity Statement

The authors are all male and represent different disciplines and levels of seniority. Four authors specialize in health economics and financing, two have extensive expertise in health systems. All the authors are locally based and have extensive experience in conducting health economics, policy and health system research in Bangladesh.

## Appendix 1

The sample size was estimated as 795 for each area considering a 30% reduction in CHE incidence among the BPL population (from 16.5% to 11.5%) as per another study (Khan *et al.*, 2017), with a 95% confidence level and 80% power. After incorporating a 1.4 design effect for stratification and village selection and a 10% non-response rate, the sample size became 1236. Based on the findings of a pilot study conducted in three different villages that yielded 14 BPL HHs seeking IPC in the last 12 months, an estimated 1260 BPL HHs (10 village × 3 strata × 3 Upazilas × 14 HHs = 1260) with IPC experience from within the past 12 months were expected to be identified from each intervention and comparison area.
