# Supplementary material for: The effectiveness of a government-sponsored health protection scheme in reducing financial risks for the below-poverty-line population in Bangladesh
Source: Health Policy Plan. 2023 Dec 20;39(3):281–98. doi: 10.1093/heapol/czad115 (PMC11423846; doi:10.1093/heapol/czad115)
Supplement: czad115_Supp [file czad115_supp.zip › supp/Appendix 1.docx]

**Appendix 1:** The sample size was estimated as 795 for each area considering a 30% reduction in CHE incidence among the BPL population (from 16.5% to 11.5%) as per another study (Khan *et al.*, 2017), with a 95% confidence level and 80% power. After incorporating a 1.4 design effect for stratification and village selection and a 10% non-response rate the sample size became 1236. Based on the findings of a pilot study conducted in three different villages that yielded 14 BPL HHs seeking IPC in the last 12 months, an estimated 1,260 BPL HHs (10 village × 3 strata × 3 Upazilas × 14 HHs =1,260) with IPC experience from within the past 12 months were expected to be identified from each intervention and comparison area.
