# Supplementary figures and images for: The effectiveness of a government-sponsored health protection scheme in reducing financial risks for the below-poverty-line population in Bangladesh
Source: Health Policy Plan. 2023 Dec 20;39(3):281–98. doi: 10.1093/heapol/czad115 (PMC11423846; doi:10.1093/heapol/czad115)

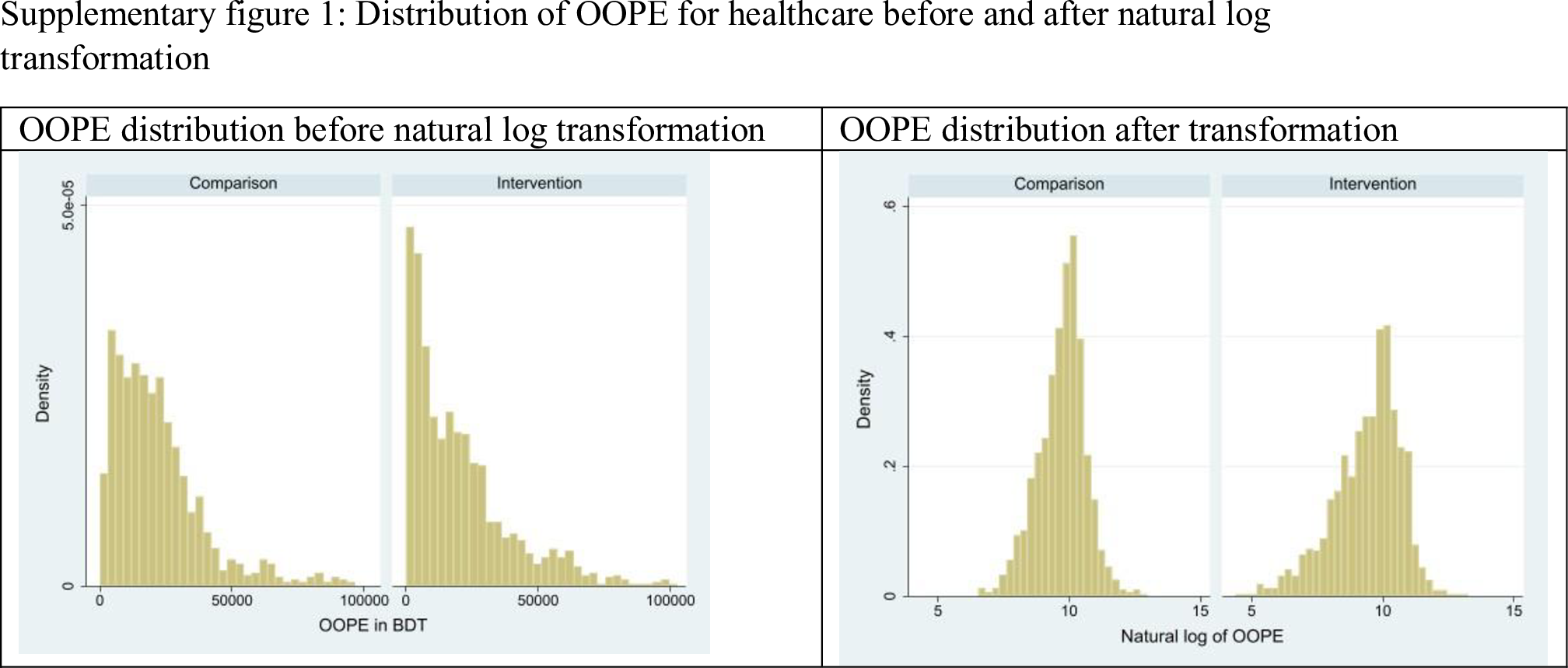

Supplement: czad115_Supp [file czad115_supp.zip › supp/Supplementary figure 1.tif]
